# Classification of Left Atrial Diseased Tissue Burden Determined by Automated Voltage Analysis Predicts Outcomes after Ablation for Atrial Fibrillation

**DOI:** 10.1155/2021/5511267

**Published:** 2021-06-22

**Authors:** Szilvia Herczeg, John J. Keaney, Edward Keelan, Claire Howard, Katie Walsh, Laszlo Geller, Gabor Szeplaki, Joseph Galvin

**Affiliations:** ^1^Heart and Vascular Centre, Semmelweis University, 68 Varosmajor Street, Budapest, Hungary; ^2^Atrial Fibrillation Institute, Mater Private Hospital, Eccles Street, Dublin 7, Ireland; ^3^Royal College of Surgeons in Ireland, Ireland

## Abstract

**Background:**

The burden and persistence of atrial fibrillation (AF) have been associated with the presence and extent of left atrial (LA) fibrosis. Recent reports have implicated an association between the extent of LA fibrosis and the outcome of pulmonary vein isolation (PVI). We aimed to analyse the value of an automated scar quantification method in the prediction of success following PVI.

**Methods:**

One hundred and nine consecutive patients undergoing PVI for paroxysmal or persistent AF were included in our observational study with a 2-year follow-up. Prior to PVI, patients underwent high-definition LA electroanatomical mapping, and scar burden was quantified by automated software (Voltage Histogram Analysis, CARTO 3, Biosense Webster), then classified into 4 subgroups (Dublin Classes I-IV). Recurrence rates were analysed on and off antiarrhythmic drug therapy (AAD), respectively.

**Results:**

The overall success rate was 74% and 67% off AAD at 1- and 2-year follow-up, respectively. Patients with Dublin Class IV had significantly lower success rates (*p* = 0.008, off AAD). Dublin Class IV (OR = 2.27, *p* = 0.022, off AAD) and the presence of arrhythmia in the blanking period (OR = 3.28, *p* = 0.001, off AAD) were the only significant predictors of recurrence. The use of AAD did not affect these results.

**Conclusions:**

We propose a classification of low voltage areas based on automated quantification by software during 3D mapping prior to PVI. Patients with high burden of low voltage areas (>31% of <0.5 mV, Dublin Class IV) have a higher risk of recurrence following PVI. Information gathered during electroanatomical mapping may have important prognostic value.

## 1. Introduction

Despite significant technological developments in the ablation of atrial fibrillation (AF) ablation, there is still a significant percentage of arrhythmia recurrence [1]. Improvements beyond ablation techniques and technology include the ability to characterise left atrial (LA) tissue conduction properties, locate fibrosis, and quantify scar burden [2]. LA fibrosis has been shown to have an important role in the mechanism of AF and can be estimated by invasive 3D electroanatomical mapping, by determining low voltage area [3]. However, in most of the studies to date, the extent of LA low voltage area is assessed by visual estimation only and not by automated, operator-unbiased measurements [4, 5]. This has been shown to overestimate the amount of dense scar and underestimates the extent of diseased atrial tissue [6]. Furthermore, there is remarkable diversity in the cut-off values applied during determining the burden of LA low voltage areas, ranging from 0.1 to 1.5 mV, with 0.5 mV being the most commonly used [6–8]. These sometimes subjective and investigator-biased methods can raise some concerns regarding the accuracy of using LA voltage information.

## 2. Aims

Recently, we have described a novel, objective, and automated method which enables rapid and accurate quantification of the Diseased LA Tissue and dense scar burden (Voltage Histogram Analysis (VHA) Tool) [9]. Our aim in the present study is to describe and propose a new classification system to assess the Diseased LA Tissue burden, based on the automated VHA tool (Dublin classification). We have also investigated the efficacy of the Dublin classification in predicting recurrent AF in patients who underwent pulmonary vein isolation (PVI).

## 3. Methods

### 3.1. Study Population

Consecutive patients were included in our study undergoing their first PVI for paroxysmal or persistent atrial fibrillation at our centre. Patients with previous left atrial ablation or with a history of any cardiac surgery were excluded. Patients were also excluded if sinus rhythm could not be maintained during voltage mapping, or if in addition to the PVI any non-PV left atrial sites were targeted. We analysed the follow-up data retrospectively to assess the success of PVI. All patients, who were enrolled in the study, gave written informed consent prior to their inclusion. The study protocol was reviewed and approved by the Local Research Ethics Committee (IRB Reference: 1/378/1909 TMR) and was in accordance with the Declarations of Helsinki.

### 3.2. Ablation Procedure and Voltage Mapping

The ablation procedure was performed in accordance with European guidelines and consensus statements. An uninterrupted oral anticoagulant strategy was used. During the procedure, IV heparin was administered with target activated clotting time between 250 and 350 seconds. Procedures were performed under general anaesthesia. Transoesophageal echocardiography was used to rule out LA thrombus and to guide the transseptal puncture. After transseptal puncture, a 20-pole diagnostic catheter was passed to the left atrium (LASSO® 2515 Eco Catheter, Biosense Webster Inc., adjustable 15 to 25 mm circumference; 2-6-2 mm interelectrode spacing). Patients with ongoing AF underwent electric cardioversion to restore sinus rhythm before mapping. In the case of hemodynamical instability, pacing from the proximal coronary sinus electrode was used during the procedure (Dynamic, Boston Scientific Inc.). Afterwards, high-density bipolar voltage mapping with the LASSO catheter was performed using the CARTO 3® platform, CONFIDENSE™ module (Biosense Webster Inc.). In order to create an accurate and detailed voltage map, the tissue proximity indication filter was turned on. All points were checked manually to exclude the ones with ventricular far field, noise, or spacing artefact. Further details of the mapping method have been previously described [9]. Next, pre-ablation cardiac computed tomography angiography (CTA) of the LA images was merged with the electroanatomical map. PVI was performed with wide antral circumferential ablation using a contact force sensor-enabled, irrigated-tip catheter (Thermocool SmartTouch, Biosense Webster Inc.). PVI was verified by demonstrating the entrance block after a 20-minute waiting period. If typical right atrial flutter was documented prior to the procedure or was recorded during the procedure, cavotricuspid isthmus ablation was performed as well.

Patients with uncomplicated procedures were discharged from the hospital the day after PVI. All patients were prescribed proton pump inhibitors for 6 weeks following the procedure, to prevent oesophageal damage. All patients continued anticoagulation for at least 2 months after the procedure.

### 3.3. Quantitative Analysis and Definition of Low Voltage Area

LA scar burden was analysed offline via the CARTO 3® VHA Tool (Biosense Webster Inc.), as previously described [9]. The pulmonary veins, mitral annulus, and left atrial appendage were manually removed and excluded from the LA voltage maps. *Dense Left Atrial Scar* was defined as any area with bipolar voltage ≤ 0.2 mV. *Diseased Left Atrial Tissue* was defined as any area with bipolar voltage ≤ 0.5 mV. The extent of Dense LA Scar and Diseased LA Tissue was expressed as percentages of the total calculated LA surface, respectively. The cut-offs for classification based on our previous publication for Dense LA Scar and Diseased LA Tissue are shown in (Table 1) and were used in this manuscript for outcome analyses. The proposed Dublin classification refers to the Diseased LA Tissue burden, with a cut-off of 0.5 mV (bipolar), where Dublin Classes I to IV represent a gradually increasing amount of low voltage areas, with Dublin Class I being the lowest and Dublin Class IV the highest.

**Figure 1:**
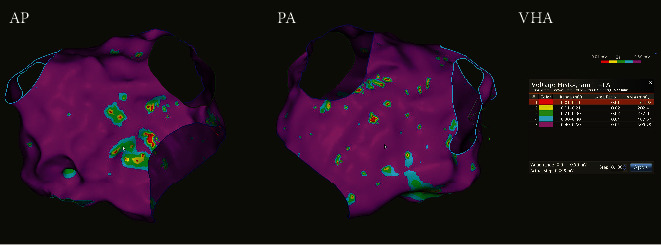
An example of presenting low voltage areas of left atria (anteroposterior (AP) and posteroanterior (PA) view) analysed by CARTO 3® Voltage Histogram Analysis (VHA) Tool. This patient classified as Class II for Dense LA Scar (2.22%) and Dublin Class II for Diseased Left Atrial (LA) Tissue (14.41%).

According to the classification shown in Table 1, this example patient had Class II Dense LA Scar and Dublin Class II Diseased LA Tissue burden (Figure 1). The Voltage Histogram shows the exact distribution of low voltage areas within the manually set ranges.

### 3.4. Follow-Up Protocol and Definition of Success

Patients were scheduled for follow-up visits at 6 weeks and 3, 6, and 12 months after the PVI. After 12 months, an annual routine follow-up was scheduled for the patients. Before every follow-up visit starting at the 3-month follow-up, a 12-lead ECG and a 24-hour Holter-ECG were conducted. In case of any new symptoms, unscheduled visits and ECG or Holter documentation of rhythm were organized. Recurrence was defined as a documented >30-second episode of atrial fibrillation or left atrial flutter/tachycardia after a 3-month long blanking period. The procedure was defined as successful, until such recurrence has been documented. A case of any symptom of supraventricular arrhythmia with or without ECG documentation during the blanking period was not considered the primary recurrence endpoint in accordance with consensus statements.

Antiarrhythmic drug (AAD) therapy was held after the blanking period; however, in some cases, regular AAD was continued or restarted at the discretion of the operator based on the patient's clinical profile (e.g., patient's symptoms, documented recurrence, or used as *pill in the pocket* therapy in cases of infrequent paroxysmal recurrences). Therefore, we have done two separate analyses on assessing the PVI success rates: (1) outcome in patients off AADs (*OFF AAD*), where recurrence was defined if the patient had documented arrhythmia (see criteria above), or AAD was administered after blanking period for any reason, even without documented evidence of arrhythmia, and (2) outcome in patients irrespective of AADs (*ON AAD)*, where recurrence was counted only in case of documented arrhythmia following the blanking period (see criteria above), irrespective of the use of AADs. Success rates of PVI based on the 2 different analyses (OFF AAD and ON AAD) are reported separately throughout the manuscript.

### 3.5. Statistical Analysis

Categorical variables were presented as even numbers and percentages and continuous variables as median and interquartile ranges. The success rate of PVI was analysed by the Kaplan-Meier survival test, compared by the log-rank test. Univariate Cox regression was performed to determine the predictors of recurrence. A two-tailed *p* value < 0.05 was considered significant. Analysis was done by Prism version 6.01 (GraphPad Software, Inc., La Jolla, CA) and IBM SPSS Statistics, Version 25 (IBM Corp., Armonk, NY) software packages.

## 4. Results

### 4.1. Patient and Procedural Characteristics

One hundred nine dominantly male (76%) patients were included with a median age of 62 [55-70] years (Table 2). Almost one-third of the patient population had persistent atrial fibrillation at the time of enrolment. Hypertension (36%), vascular disease (15%), and underlying heart disease (15%) were the most frequent comorbidities. Seven percent of the patients had left ventricular ejection fraction (LVEF) < 50%.

During the ablation procedure, median 958 [658-1257] points were collected while mapping the LA during median 10 [8-14] minutes. All patients were classified: (1) by the extent of LA Dense Scar (≤0.2 mV) and (2) by the extent of LA Diseased Tissue (≤0.5 mV) by Dublin Classes I-IV (Table 3.)

Acute success of the PVI, demonstrated by entrance block to the veins, was achieved in all cases. Five (4.6%) major complications were recorded (3 pericardial effusion requiring pericardial drainage, 1 severe pericarditis, and 1 right phrenic nerve palsy), and these were all unrelated to the mapping process and the VHA. These resolved without sequelae.

### 4.2. Follow-Up Results and Overall Success Rates

**Figure 2:**
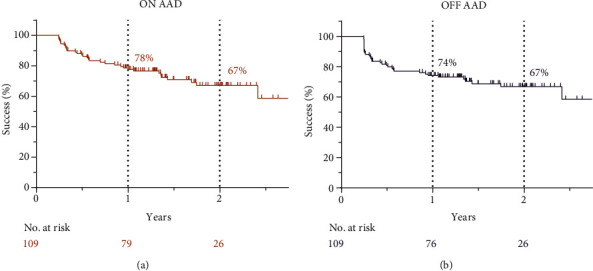
Overall success of first pulmonary vein isolation for atrial fibrillation ON AAD (a) and OFF AAD (b).

The median duration of the follow-up period was 632 [469-760] days. We detected 30 (28%) patients having symptoms of palpitation or supraventricular arrhythmia during the blanking period with or without ECG documentation. After the blanking period, 23 (21%) patients used AADs. There were 33 (30%) cases of recurrence at the ON AAD analysis, and 35 (32%) recurrences at the OFF AAD analysis. Out of the 25 patients with paroxysmal AF, who had a recurrence, 18 (72%) patients continued to have paroxysmal AF and 5 (20%) progressed to the persistent stage. Two patients (4%) experienced no recurrence; however, they stayed on AADs up to 4 months after PVI, thus reaching the set endpoint. Out of the 10 patients who had persistent AF before ablation and experienced a recurrence, 8 (80%) developed persistent AF, and in 2 cases (20%) we observed an improvement to a paroxysmal type of AF postablation. Twelve (9%) patients underwent electrical cardioversion, and 24 (22%) patients underwent repeat ablation. The overall success rate was 78% and 67% ON AAD, and 74% and 67% OFF AAD at the 1- and 2-year follow-up, respectively (Figure 2).

### 4.3. The Correlation of Left Atrial Low Voltage Area with the Success Rates

**Figure 3:**
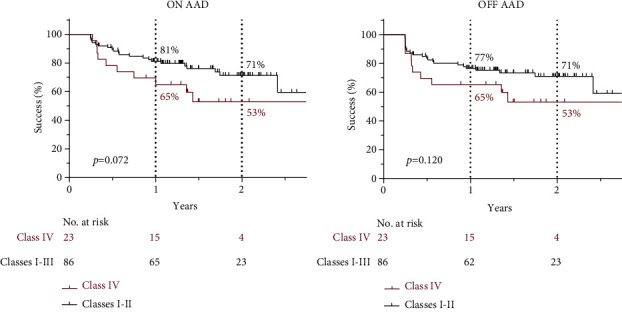
Success rate of patients with Class IV vs. Classes I-III Dense Left Atrial Scar (log-rank test).

The success rates of PVI in the different Dense LA Scar (≤0.2 mV) Classes were compared. There was no statistically significant separation of the survival curves in Classes I-III, but Class IV showed a tendency to lower success rates (*p* = 0.207 ON AAD or *p* = 0.388 OFF AAD, log-rank test). Therefore, Classes I-III were grouped together and compared with Class IV. Although there was a tendency, we did not find a significant difference in success rates between Dense LA Scar Classes I-III versus Class IV patients (*p* = 0.072 ON AAD or *p* = 0.120 OFF AAD, Figure 3).

**Figure 4:**
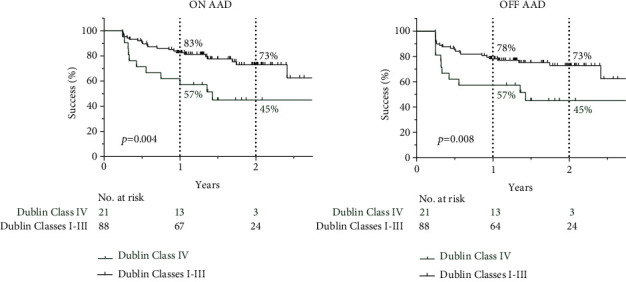
Success rate of patients with Dublin Class IV vs. Dublin Classes I-III Diseased Left Atrial Tissue (log-rank test).

In the Diseased LA Tissue burden (≤0.5 mV) analysis, there was a statistically significant separation of the four survival curves, and Dublin Class IV showed the lowest success rate (*p* = 0.016 ON AAD or *p* = 0.031 OFF AAD, log-rank test). Similarly, Dublin Classes I-III were grouped together and compared with Dublin Class IV. Patients with Dublin Class IV had a significantly lower success rate than those with Dublin Class I-III, irrespective of AAD use (*p* = 0.004 ON AAD or *p* = 0.008 OFF AAD, Figure 4). A similar trend was observed in the subgroup of patients with paroxysmal (log-rank analysis, *p* = 0.049) and persistent (log-rank analysis, *p* = 0.065) AF.

### 4.4. Predictors of Recurrence

Afterwards, we determined the predictors of recurrence in both ON AAD and OFF AAD analyses, respectively (Table 4). Among the various clinical and procedural parameters, only two significant predictors were found: the presence of arrhythmia in the blanking period (OR = 3.14, *p* = 0.001 for ON AAD; OR = 3.28, *p* = 0.001 for OFF AAD) and Dublin Class IV (OR = 2.51, *p* = 0.012 for ON AAD; OR = 2.27, *p* = 0.022 for OFF AAD, Table 4). No other parameters predicted recurrence in the present study.

## 5. Discussion

### 5.1. Role of Left Atrial Fibrosis

Increasing evidence suggests the importance of atrial fibrosis in the presentation and progression of AF. Atrial fibrosis is a significant contributor to the complex pathomechanism of atrial fibrillation, and they together act in a vicious circle, while the arrhythmia itself can lead to “structural, architectural, contractile, or electrophysiological changes” in the atria, and the fibrotic changes contribute to the manifestation of AF [10]. Histological examinations of atrial biopsy samples have shown that in patients with persistent lone AF, more severely remodelled atrial tissue and interstitial fibrosis are found, compared to subjects without any known heart disease [10, 11]. Currently, various clinical tools based on electrocardiography, electrophysiology, echocardiography, and cardiac MRI are available to estimate the severity of atrial fibrosis [12–16]. It seems that with the progression of atrial fibrosis, the treatment of AF becomes more challenging and the arrhythmia-free survival is shortened.

### 5.2. Characterisation of Low Voltage Area

An increasing number of studies have examined the role of LA low voltage areas (LVAs) in the maintenance of sinus rhythm after ablation for AF. The method of estimating the extent of LVA in most of the cases is not automated and mainly depends on visual estimation; therefore, operator bias may be a significant limitation of these methods [6]. In the majority of the studies, LVAs are estimated based on the presence of ≥3 adjacent scarred points (peak-to-peak bipolar voltage electrogram), which are < 3 mm apart from each other [4, 7, 17–19]. Mapping is typically performed with the use of a multipolar mapping catheter and completed with point-by-point mapping using the ablation catheter. The most important advantage of the mapping method we have used in our study is that it allows more exact and unbiased quantification of LVAs, based on an automated software algorithm [9]. The analysis of the extent of LA LVAs by VHA is based on the actual area defined by LVAs in a triangulated mash model and might be more accurate than counting the number of low voltage points only [9].

There is no consensus in the literature regarding the cut-off values defining LVAs and also in the cut-offs for defining the groups of fibrosis severity used in the prediction of outcomes [4, 17, 20]. In general, the definition of LVAs are typically ≤0.5 mV bipolar (0.2 mV and 0.4 mV have also been used). Regarding the definition of severity, the most simplistic approach of classification is based on the presence or absence of the LVAs, while the other commonly used classification uses four different stages (i.e., none, mild, moderate, and severe) [4, 17, 20]. Our predefined classification cut-offs for the LVAs were (1) 0.2 mV, which was termed as Dense LA Scar, and (2) 0.5 mV, which we referred to as Diseased LA Tissue. For the definition of the cut-offs of the different severity subgroups, we used a purely mathematical approach by defining the quartiles based on the first 100 patients' data [9].

### 5.3. Predictors of Recurrence Post Pulmonary Vein Isolation

When analysing the data on the success of PVI in the present cohort, we were able to show that there is a clear association between the extent of Diseased LA Tissue (which we refer to as Dublin Classification based on the cut-offs and criteria above, Table 3) and the success of PVI. Patients in Dublin Class IV have a significantly higher chance of recurrence after PVI. No such observation was made with the Dense LA Scar classification in our cohort. The type of atrial fibrillation (paroxysmal versus persistent) was not a predictor of recurrence in the present cohort. It seems that though persistent atrial fibrillation patients have a higher low voltage area burden and their overall success rate is lower compared with paroxysmal AF patients, still the high amount of scar is what is associated with the unfavourable outcomes [5, 6, 9]. Our findings are in line with previous observations, which suggests that the scar burden might be a more reliable predictor of success of AF ablation, than the binary classification of AF based on episode duration.

Outcomes from previous studies have shown a similar association between high LVA burden and recurrence after catheter ablation of AF; however, the methods are not uniform. Wang et al. manually contoured the identified LVA (<0.5 mV) on a 3D map of 150 patients with a contact force sensing single-tip ablation catheter (150-200 points collected). The percentage of LVA of the total LA surface was calculated by CARTO® software, and they categorized the patients into four subgroups: none (0%), mild (<10%), moderate (10-20%), and severe (>20%). According to their results, patients with >10% LVA had significantly higher risk for AF recurrence [18]. Vlachos et al. included 80 patients in their study with a higher resolution of the maps (2485 points median) and used four categories as well: <10%, 10-20%, 20-30%, and >30% of LVA with 2 criteria for LVA definition. According to their data, >10% of <0.4 mV LVA was the only predictor for AF recurrence [7]. It seems that there is a consistency, and the high burden of LVA is a predictor of AF recurrence. However, in our study, only patients with the highest quartile of LVA burden (>31% of ≤0.5 mV) showed inferior outcome, and no other conventional baseline clinical parameters predicted the success of AF ablation. This later observation is in line with Vlachos et al, while Huang et al. found that higher CHA_2_DS_2_-VASc score, LA volume, and AF type are also risk predictors for recurrence [7, 19].

In our studied population, the one- and two-year success rates were 57% and 45% for Dublin Class IV vs. 78% and 73% for Dublin Classes I-III OFF AAD, while 57% and 45% for Dublin Class IV vs. 83% and 73% for Dublin Classes I-III with the ON AAD analysis, respectively. Our follow-up method was similar to those used in the literature; therefore, outcomes between the studies are comparable. Most studies report their outcomes OFF AAD; however, as there is inconsistency in that regard, we decided to show outcomes OFF AAD and ON AAD as well. Based on the literature data, our success rates were within the previously reported ranges: 72-93% at one year with low LVA and 28-75% at one year for high LVA burden [4, 7, 17, 18, 20].

### 5.4. Main Findings

Previously, we have described that patients with the highest Diseased LA Tissue burden (Dublin Class IV) were older, more likely female, had higher CHA_2_DS_2_-VASc scores, and presented more likely with persistent AF [9]. We have shown that Dublin Class IV predicts the success of PVI in patients with AF. In line with the literature, arrhythmia in the blanking period was also revealed to be a risk factor for recurrence, but none of the baseline clinical covariates predicted the recurrence of atrial fibrillation [21]. According to our results, Dense LA Scar burden is not a good predictor of AF recurrence. The arrhythmia-free success rates were similar for ON and OFF AAD analysis, and the use of AAD did not relevantly affect outcomes at 2 years. The Voltage Histogram Analysis Tool is an automated, quick, reproducible, and reliable software to assess precisely the amount of LA low voltage area. It seems that VHA identifies patients at high risk for recurrence at the time of their ablation. This finding may help to develop a more personalized management for patients after their pulmonary vein isolation by adjusting the frequency of follow-up visits and managing the patient's expectations. There might be an increased need for additional rhythm control therapies by further catheter ablation using different non-PV targets in this subgroup of patients; however, further studies will have to confirm the potential benefit of such an approach.

## 6. Limitations

The most important limitation of our single centre study is the relatively low number of patients included; however, the overall number of the population is comparable to those reported in the literature. Currently, the VHA tool is not commercially available, and therefore, its use is limited to some centres only. Even though we used all tools to avoid poor contact during mapping, it might have affected the maps for a certain degree and lead to underestimation of voltage. Manual exclusion of left atrial appendage, pulmonary veins, and mitral annulus on the bipolar voltage maps represents a potential operator bias. Information on the exact duration of recurrent atrial fibrillation episodes was not collected in this study.

## 7. Conclusion

In our study, 109 patients underwent high-density bipolar voltage mapping before pulmonary vein isolation for paroxysmal and persistent atrial fibrillation. Offline analysis was made by a novel, automated, and quantitative Voltage Histogram Analysis Tool to quantify the percentage of low voltage areas of the left atrium. Patients were classified into four subgroups based on the severity of Diseased Left Atrial Tissue (≤0.5 mV) and Dense Left Atrial Scar (≤0.2 mV). During the two-year long follow-up period, the patients with the most extensive Diseased Left Atrial Tissue burden (Dublin Class IV) developed more recurrences. Arrhythmia in the blanking period, but no other baseline clinical parameters predicted the success of pulmonary vein isolation. Application of this classification may be a useful clinical tool in predicting recurrent atrial fibrillation post pulmonary vein isolation.

## Figures and Tables

**Table 1:** Classification of Dense Left Atrial (LA) Scar (Classes I-IV) and Diseased LA Tissue (Dublin Classes I-IV), where percentages show the extent of the LA low voltage areas (≤0.2 and ≤0.5 mV, respectively).

Dense LA Scar (≤0.2 mV)		Diseased LA Tissue (≤0.5 mV)	
Class I	<1%	Dublin Class I	<9%
Class II	1%–3%	Dublin Class II	9%–18%
Class III	3%–8%	Dublin Class III	18%–31%
Class IV	>8%	Dublin Class IV	>31%

LA: left atrium.

**Table 2:** Baseline and procedural characteristics.

Baseline and procedural characteristics (*n* = 109)		
Gender (female)	26	(24%)
Median age (years)	62	[55-70]
Median BMI (kg/m^2^)	28	[26-31]
Persistent AF	34	(31%)
CHA_2_DS_2_-VASc score ≥ 2	41	(38%)
LVEF <50%	8	(7%)
Hypertension	39	(36%)
Diabetes mellitus	0	(0%)
Prior stroke or TIA	5	(5%)
Vascular disease	16	(15%)
Coronary artery disease	10	(9%)
Underlying heart disease	16	(15%)
Prior CTI ablation	8	(7%)
Median LAV (CTA) (ml)	142	[124-169]
Median no. of mapped LA points	958	[658-1257]
Mapping time (min)	10	[8-14]
Dense LA Scar (≤0.2 mV) (%)	2.49	[0.54-7.17]
Diseased LA Tissue (≤0.5 mV) (%)	15.85	[6.94-28.41]

AF: atrial fibrillation; BMI: body mass index; CTA: computed tomography angiography; CTI: cavotricuspidal isthmus; LA: left atrium; LAV: left atrial volume; LVEF: left ventricular ejection fraction; TIA: transient ischaemic attack.

**Table 3:** Distribution of the study population in the Dense Left Atrial Scar and Dublin Classes.

Dense LA Scar (≤0.2 mV)	No. patients (%)		Diseased LA Tissue (≤0.5 mV)	No. patients (%)	
Class I	36	(33%)	Dublin Class I	36	(33%)
Class II	21	(19%)	Dublin Class II	25	(29%)
Class III	29	(27%)	Dublin Class III	27	(25%)
Class IV	23	(21%)	Dublin Class IV	21	(19%)

LA: left atrium.

**Table 4:** Univariate Cox regression test for predictors of recurrence (separate ON AAD and OFF AAD analyses).

Parameters	*n* (%)		ON AAD analysis			OFF AAD analysis		
			*p*	OR	CI (95%)	*p*	OR	CI (95%)
Gender (female)	26	(24%)	0.94			0.96		
BMI > 30 kg/m^2^	25	(23%)	0.73			0.35		
Age ≥ 65 years	41	(38%)	0.28			0.47		
Persistent AF	34	(31%)	0.90			0.62		
CHA_2_DS_2_‐VASc ≥ 2	41	(38%)	0.32			0.53		
LVEF <50%	8	(7%)	0.12			0.25		
Hypertension	39	(36%)	0.97			0.81		
Diabetes mellitus	0	(0%)	—			—		
Prior stroke or TIA	5	(5%)	0.11			0.12		
Vascular disease	16	(15%)	0.22			0.17		
Coronary artery disease	10	(9%)	0.74			0.61		
Underlying heart disease	16	(15%)	0.70			0.92		
LAV > 42 ml (CTA)	56	(51%)	0.27			0.27		
Arrhythmia in blanking period	30	(28%)	0.001	3.14	1.55-6.36	0.001	3.28	1.65-6.52
Class IV	23	(21%)	0.10			0.17		
Dublin Class IV	21	(19%)	0.012	2.51	1.22-5.14	0.022	2.27	1.12-4.61

AAD: antiarrhythmic drug; AF: atrial fibrillation; BMI: body mass index; CTA: computed tomography angiography; LVEF: left ventricular ejection fraction; LAV: left atrial volume; TIA: transient ischaemic attack.

## Data Availability

The data that support the findings of this study are available from the corresponding author upon reasonable request.
